# Contribution of galvanic vestibular stimulation for the diagnosis of HAM/TSP

**DOI:** 10.1186/1742-4690-11-S1-P27

**Published:** 2014-01-07

**Authors:** Luciana Cunha, Ludimila Labanca, Anna Bárbara F Carneiro-Proietti, Lucas N Carvalho, Daniele R Fernandes, Ana Lúcia B Starling, Denise U Gonçalves

**Affiliations:** 1 HTLV Interdisciplinary Research Group (HTLV-1), Belo Horizonte, Minas Gerais, Brazil; 2 Tropical Medicine Post Graduation Program, Faculty of Medicine, Federal University of Minas Gerais, Belo Horizonte, Minas Gerais, Brazil; 3 Foundation and Center for hematology and hemotherapy of Minas Gerais, Belo Horizonte, Minas Gerais, Brazil

## Introduction

Galvanic vestibular stimulation (GVS) is a low-cost and safe exam that tests the vestibulospinal pathway. HTLV-1-associated myelopathy/tropical spastic paraparesis (HAM/TSP) is a slowly progressive disease that precociously affects the vestibulospinal tract. This study compared electromyographic (EMG) responses triggered by GVS between asymptomatic individuals infected with HTLV-1 and those with HAM/TSP.

## Methods

Bipolar galvanic stimuli of 400 ms and 2 mA intensity were applied on the mastoid processes in 120 trials. The EMG response, and its short latency (SL) and middle latency (ML) components, was recorded from both soleus muscles in 13 healthy, HTLV-1-negative adults (56 ± 5 years, mean ± SD), and 26 individuals infected with HTLV-1, 13 asymptomatic (56 ± 8 years) and 13 with HAM/TSP (60 ± 6 years).

## Results

The average value of EMG components in the group of healthy individuals was 55 ± 4ms (SL) and 112 ± 10ms (ML); in the group of asymptomatic HTLV-1 carriers, 61 ± 6ms (SL) and 112 ± 10ms (ML); and in the group with HAM/TSP, 67 ± 8ms (SL) and 130 ± 3ms (ML) (P=0.001). In the HTLV-1-asymptomatic group, the SL component was delayed in 4/13 (31%) exams and the ML component was normal in all. In the HAM/TSP group, the most common alteration was the absence of waves.

## Conclusion

A pattern of abnormal vestibular-evoked EMG response was found in the HTLV-1-neurological disease, which ranged from delayed latency among asymptomatic carriers to absence of response in HAM/TSP. GVS may contribute to early diagnosis and monitoring of non-traumatic myelopathies.

